# The Acari Hypothesis, VIII: the human apocrine–mammary–microbiota axis and *Staphylococcus epidermidis* mutualism

**DOI:** 10.3389/fmicb.2026.1714798

**Published:** 2026-05-28

**Authors:** Andrew C. Retzinger

**Affiliations:** Department of Emergency Medicine, Camden Clark Medical Center, West Virginia University, Parkersburg, WV, United States

**Keywords:** apocrine glands, human evolution, mammalian evolution, mammary glands, skin, *Staphylococcus epidermidis*, the Acari Hypothesis

## Abstract

Human integument is unusual among primates for its expanded eccrine network, glandular specialization, acidic surface pH, and enrichment of staphylococcal taxa. Framed within the Acari Hypothesis, this manuscript proposes a unifying apocrine-mammary-microbiota axis in which human integumentary glands cultivate protective symbionts, with *Staphylococcus epidermidis* as a keystone. Reviewed evidence encompasses acid mantle biochemistry and glandular antimicrobials that favor acid- and lipid-tolerant staphylococci while restricting pathogens and possibly ectoparasites. Mammalian apocrine secretions rely on microbial metabolism to convert precursor molecules into bioactive compounds that mediate species-specific physiological functions. This reliance may be consistent with the enrichment of staphylococci at apocrine-rich sites in humans. Mammary biology indicates vertical transfer of skin- and milk-associated microbes that stabilize epithelial ecosystems across generations. The manuscript develops a mutualist model for *S. epidermidis* on human epithelia, reconsiders device-associated infections, and surveys staphylococcal dysbiosis across dermatologic conditions. As proposed, human integumentary and mammary glands co-evolved to cultivate and vertically transmit symbiotic staphylococci, whose metabolites and niche competition defend against ectoparasites and pathogens while balancing epithelial and metabolic homeostasis.

## 1 Introduction

The Acari Hypothesis proposes that acarians (mites and ticks) may have exerted persistent selective pressure on mammals, shaping epithelial structure and function (Retzinger and Retzinger, 2020, 2021, 2022, 2024a,b,c, 2025). Adaptations attributed to this influence include the emergence of immunoglobulin E (IgE), a uniquely mammalian antibody associated with epithelial defense, and the transformation of the integument from a predominantly keratinized barrier into one enriched with sebaceous, eccrine, and apocrine glands. The defensive function of mammalian integument extends beyond the intrinsic antimicrobial properties of glandular secretions to include the ecological niches these glands create for symbiotic microorganisms. As an example, sebaceous glands sustain fatty acid-dependent *Malassezia* spp., whose colonization may contribute to rendering mammalian skin inhospitable to acarians (Retzinger and Retzinger, 2024b,c). Here, gland-supported microbes may provide anti-acarian protection.

The proposed symbiosis between sebaceous glands and *Malassezia* spp. may illustrate a broader principle of mammalian physiology: integumentary glands actively cultivate beneficial microbial communities, and these communities in turn confer advantages to the host. This adaptive capacity, recruiting site- and species-specific microbes, may represent an underappreciated evolutionary strategy, offering defenses against ectoparasites, pathogens, and other environmental challenges.

Among mammals, the integument of catarrhine primates is notable for its glandular specialization, particularly the expanded density and distribution of eccrine glands (Best and Kamilar, 2018). Unlike most mammals, which primarily restrict eccrine glands to palmar and plantar skin, catarrhines exhibit a broader distribution. In humans, this trend is especially pronounced: eccrine glands occur at high densities across much of the body, ∼500/cm^2^ on the palms and soles, ∼200/cm^2^ on the scalp and face, and ∼100/cm^2^ in the axillae (Taylor and Machado-Moreira, 2013). Human apocrine glands are concentrated in the axillae, nasal vestibule, and anogenital region, including the inguinal folds, prepuce, external labia, and perianal skin, while sebaceous glands are most densely distributed on the face and scalp (400–900/cm^2^) (Ludovici et al., 2018). This pattern reflects a marked departure from that of other mammals, including other primates.

Humans also diverge from other primates in their associations with acarian ectoparasites. Other primates host obligate skin-dwelling mites of the family Psoroptidae, which feed directly on living tissue (Bochkov and Grootaert, 2014; Bochkov et al., 2010; Bochkov, 2011). In contrast, humans are not routinely infested by psoroptid mites, although they do commonly harbor *Demodex* spp. and *Sarcoptes scabiei*, distinct lineages. Instead, human environments are largely shared with Pyroglyphidae (dust mites), sister clade to Psoroptidae, which subsist on shed epidermal debris (Klimov and OConnor, 2013). This contrast raises the possibility that adaptations of human integument, together with its distinct cutaneous microbiota, may have evolved to deter psoroptid mites. If so, this shift could help explain subsequent eccrine expansion.

Consistent with the view that glandular anatomy shapes the mammalian cutaneous microbiota, human skin harbors a distinct profile enriched in staphylococcal taxa. Comparative analysis of primate axillary microbiota highlights this specialization: *Staphylococcus* comprises roughly 47% of the human axillary community, compared with only 8% in chimpanzees and 6% in gorillas (Council et al., 2016). Within humans, staphylococci show a preference for the moist microenvironments created by apocrine and eccrine secretions (Joglekar et al., 2023). Amplicon-based microbial profiling estimates their relative abundance at ∼30% in the inguinal crease, ∼10% on the palms, ∼60% on the soles, and ∼14% on the face (Joglekar et al., 2023). Among staphylococcal species, *Staphylococcus epidermidis* often predominates, accounting for most isolates (Joglekar et al., 2023). Yet this dominance masks substantial intraspecies diversity: individual humans typically carry multiple *S. epidermidis* strains derived from 12 to 20 founder lineages (Joglekar et al., 2023). This variability likely contributes to the stability of *S. epidermidis* communities and their adaptation across skin sites.

Staphylococcal colonization is enriched where eccrine and apocrine glands co-occur, implicating apocrine secretions in sustaining these populations (Kloos and Musselwhite, 1975; Troccaz et al., 2015). The placement of these glands may, in turn, reflect an evolved defensive strategy against ectoparasites. In line with this view, the Acari Hypothesis has proposed that expansion of the eccrine system arose as a countermeasure to galactose-α-1,3-galactose (α-Gal) hypersensitivity, an acarian-mediated disease (Retzinger and Retzinger, 2024a). Eccrine glandular expansion not only increased the dispersion of secretions across the skin surface but also potentially facilitated greater staphylococcal abundance, raising the possibility that protection derives not from secretions alone but from the *Staphylococcus* spp. they sustain.

While the Acari Hypothesis has considered the roles of the eccrine and sebaceous gland in human anti-acarian defense, the roles of the apocrine gland and of the mammary gland, the apocrine gland’s proposed evolutionary derivative (Oftedal, 2002), remain largely unexplored. Apocrine glands produce viscous, protein- and lipid-rich secretions and are broadly conserved across mammals, though their distribution and function vary widely among species. In many nonhuman mammals, apocrine secretions mediate chemical communication (Fontani et al., 2022; Janssenswillen et al., 2021; Kioko et al., 2017; Leclaire et al., 2017; Ma et al., 2022; Svendsen and Jollick, 1978; Theis et al., 2012). Importantly, these secretions are initially odorless and require metabolism by resident microbes to yield signaling molecules. This dependence implies a prerequisite for apocrine function: a microenvironment that selects microbes capable of converting secretions into bioactive signals. Although chemical signaling is attenuated in humans, this more primitive function, supporting useful microbes, may be preserved. Given the frequent abundance of *S. epidermidis* in apocrine-rich skin, a plausible role for apocrine glands in humans might be to maintain stable *S. epidermidis* communities.

The theoretical dependence of humans and other mammals on microbes for signaling and cutaneous defense raises an additional biological question: how do microbe-naïve offspring acquire this microbiota? In this context, the mammary gland emerges as a likely conduit. Mammary secretions carry microbes derived from maternal epithelial surfaces (Dombrowska-Pali et al., 2024; Keady et al., 2023). Nursing facilitates the vertical transfer of these microbes from mother to offspring (Azevedo et al., 2023; Fehr et al., 2020). Within this framework, the proposed evolutionary derivation of the mammary gland from the apocrine gland is congruent. The mammary gland extends apocrine functionality by ensuring the continuity of beneficial microbial partnerships across generations. Consistent with this, *S. epidermidis*, a common colonizer of apocrine-rich skin, is frequently reported as the most abundant bacterium in human mammary secretions (Lee et al., 2024; Miura et al., 2023).

Collectively, these observations suggest the need for a broader evolutionary framework. The Acari Hypothesis, developed across prior installments in this series, proposes that acarian ectoparasites have acted as a significant selective pressure in mammalian evolution. However, this perspective is not yet widely adopted within evolutionary biology, and alternative explanations should be acknowledged.

Several of the traits considered here, including the expansion of eccrine sweating in catarrhine primates, reduction of body hair, and shifts in cutaneous microbial composition, are more commonly interpreted through the lens of thermoregulation, particularly in relation to diurnal activity and heat dissipation in open environments (Best and Kamilar, 2018). These explanations are well-supported and likely capture important components of the selective landscape.

However, thermoregulatory models do not fully account for several associated patterns, including the consistent enrichment of antimicrobial staphylococcal species on human skin, the apparent loss or reduction of psoroptid ectoparasites in humans relative to other primates, and the coordinated evolution of glandular secretions with antimicrobial properties. The Acari Hypothesis is therefore not proposed as a replacement for thermoregulatory explanations, but as a complementary and potentially unifying framework. By incorporating host-parasite dynamics alongside thermal pressures, it offers a more comprehensive account of how multiple integumentary traits, including sweating, sebum composition, microbial symbiosis, and ectoparasite resistance, may have co-evolved under overlapping selective forces.

In addition to its relevance for integumentary evolution, this framework may also help to organize several otherwise disconnected observations in human immunology and disease pathogenesis. As introduced above, IgE may be interpreted as an antibody class specialized for defense against ectoparasitic vectors, rather than solely as a maladaptive byproduct (Retzinger and Retzinger, 2021). The hypothesis also contextualizes the modern rise of IgE-mediated disease within the loss of ancestral protective factors, including glandular secretions and secretion-dependent microbial communities, under conditions of hygiene and ecological disruption (Retzinger and Retzinger, 2024a).

This perspective provides a potential explanation for the age distribution of allergic disease, linking its predominance in childhood to the delayed maturation of sebaceous gland activity and the subsequent establishment of lipid-dependent microbial communities such as *Malassezia* (Retzinger and Retzinger, 2024c). It also offers a framework for understanding the molecular selectivity of allergic responses, which disproportionately target a limited set of protein families (Retzinger and Retzinger, 2024b). Finally, it provides a basis for the comorbidity of allergic disease with conditions such as metabolic syndrome, interpreting both as consequences of disrupted epithelial ecosystems that permit colonization by invasive or opportunistic species (Retzinger and Retzinger, 2025).

Taken together, these considerations suggest that incorporating ectoparasite-driven selection into existing models may improve the explanatory scope of current frameworks, particularly in areas where thermoregulatory or purely environmental explanations remain incomplete.

The apocrine–mammary–microbiota axis proposed in this installment has implications for mammalian evolution and human health. On an evolutionary timescale, mammary gland–mediated cohabitation with microbiota may have promoted co-evolution and mutualism. In this context, the relative abundance of *S. epidermidis* on human apocrine-rich epithelial surfaces and in human mammary secretions suggests a mutualistic role in which human epithelia provide a favorable environment while the bacterium confers benefits including defense against pathogens and ectoparasites. Modern practices may disrupt this relationship. Synthetic detergents alter epithelial secretions in ways that reduce habitat suitability, while broad-spectrum antibiotics diminish populations established early in life.

## 2 *S. epidermidis* and the acidic cutaneous microenvironment of humans

The acidity of human skin differs from that of other mammals, including other primates. Whereas other mammals maintain a more neutral skin surface, human skin is distinctly acidic, with a pH of 4.1–5.8 (Proksch, 2018). This “acid mantle” inhibits pathogen proliferation and selects for acid-tolerant commensals such as *S. epidermidis* (Brooks et al., 2025). Human cutaneous acidity arises from the coordinated contributions of keratinocytes and integumentary glands.

Keratinocytes participate in the acidification of the stratum corneum through multiple mechanisms. These include lactic acid production via glycolysis, secretion of lamellar body lipids that are metabolized into fatty acids, and direct proton extrusion mediated by the sodium–hydrogen exchanger 1 (NHE1) (Fluhr et al., 2001, 2004; Ruan et al., 2025). An additional critical pathway involves filaggrin catabolism. During terminal differentiation, filaggrin is enzymatically degraded into amino acids such as histidine and glutamine, which are subsequently converted into urocanic acid and pyrrolidone carboxylic acid (Elias, 2017; Gruber et al., 2011). Together, these processes help to maintain the low surface pH of human skin.

Integumentary glands reinforce skin acidity through specialized components of their secretions. Eccrine glands secrete hypotonic sweat containing lactic acid, amino acids, and chloride, with proton-coupled monocarboxylate transporters facilitating lactic acid export and NHE1 activity adding protons directly to the surface (Baker and Wolfe, 2020). Sebaceous glands release sebum rich in triglycerides, wax esters, and squalene (Camera et al., 2010). Host and microbial lipases hydrolyze these triglycerides into free fatty acids such as palmitic and sapienic acid, which lower pH (Marples et al., 1970; Puhvel et al., 1975; Ro and Dawson, 2005). Apocrine glands produce lipid- and protein-rich secretions that are metabolized by skin microbiota into short-chain fatty acids, e.g., acetic and propionic acid, contributing to localized acidification (Mogilnicka et al., 2020). Together, these glandular outputs lower pH and further strengthen the acid mantle. Consistent with the comparative patterns above, the acid mantle favors *Staphylococcus* spp., with *S. epidermidis* frequently abundant in human skin surveys, though estimates vary by sampling method (Costello et al., 2009; Grice et al., 2009; Human Microbiome Project Consortium, 2012; Oh et al., 2014).

*S. epidermidis* withstands the acidity of human skin through complementary strategies that neutralize protons and remodel the bacterial cell surface. First, ammonia-generating pathways buffer the local milieu: the arginine deiminase (ADI) pathway yields both ATP and ammonia, and, in urease-positive strains, urea hydrolysis adds further ammonia and CO2, together elevating pH in the immediate microenvironment (Lindgren et al., 2014). Second, exposure to acidity drives metabolic remodeling, with increased synthesis of peptidoglycan and lipoteichoic acids (LTAs) that stabilize the surface and limit proton ingress (Gonçalves et al., 2022). These traits confer a significant ecological advantage over less acid-tolerant bacteria such as *S. aureus* (Iyer et al., 2021), enabling *S. epidermidis* to remain metabolically active and maintain a stable, protective niche on the human epidermis.

As will be discussed, beyond acidity, glandular secretions impose additional, species-specific constraints on colonizers. The ability of *S. epidermidis* to overcome these challenges and thrive in the uniquely harsh microenvironment of human skin may reflect a co-evolved relationship with human epithelia.

## 3 Human integumentary glands

### 3.1 Eccrine glands

Eccrine glands are a defining feature of human skin, and their secretions pose a substantial barrier to many colonizing microbes. These simple, coiled tubular structures secrete sweat by a merocrine mechanism, producing a hypotonic fluid of water, electrolytes, and immune effector molecules (Baker, 2019; Schittek et al., 2001). Humans possess approximately 2–4 million eccrine glands distributed broadly across the body, with the highest densities on the palms, soles, and forehead (Baker, 2019). Unlike other mammalian skin appendages, eccrine glands open directly onto the skin surface, independent of hair follicles. Although thermoregulation is a primary function, emerging evidence indicates that eccrine secretions also contribute to cutaneous immunity by modulating pH and ionic composition and by delivering potent antimicrobial peptides (AMPs) (Csõsz et al., 2015). Dermcidin, constitutively produced by eccrine dark cells, is a key AMP in this system (Rieg et al., 2004; Schittek et al., 2001).

The Acari Hypothesis proposes that the expansion of eccrine glands in catarrhine primates arose as an evolutionary response to selective pressures from ectoparasitic acarians (Retzinger and Retzinger, 2024a). This is consistent with two observations: First, the pathogenesis of the α-Gal hypersensitivity response, an acarian-induced pathology (Commins et al., 2011), and second, the overlap in phylogenetic timing between catarrhine *GGTA1* inactivation (encoding α1,3-galactosyltransferase, eliminating α-Gal expression) and body-wide increases in eccrine gland density and distribution (Best and Kamilar, 2018; Galili et al., 1987). Within this framework, eccrine glands may contribute to anti-acarian immunity directly, via antimicrobial or anti-acarian activities (e.g., dermcidin acting on acarians or their symbionts) and/or indirectly by fostering protective microbes such as *S. epidermidis*.

Although direct anti-acarian activity of dermcidin has not been demonstrated, its unique presence in catarrhine eccrine secretions and the evolutionary timing of gland expansion suggest that it could contribute to defenses relevant to α-Gal hypersensitivity. Unlike many cationic antimicrobial peptides, dermcidin is anionic (Schittek et al., 2001). It operates by forming zinc-dependent ion-channel assemblies, disrupting the membrane potential and leading to cell death (Paulmann et al., 2012). It remains active across a wide pH range, making it well suited to the acidic environment of human skin (Paulmann et al., 2012). Dermcidin exhibits broad-spectrum antimicrobial activity against numerous pathogens, including *S. aureus*, *Escherichia coli*, and *Mycobacterium tuberculosis* (Lai et al., 2005; Schittek et al., 2001). It may then help shape the microbial composition of the human cutaneous microenvironment.

Despite sensitivity to dermcidin under sweat-like pH and salt conditions, *S. epidermidis* persists in eccrine-rich niches by reducing peptide activity through protease-mediated inactivation and biofilm-matrix protection (Lai et al., 2007; Paulmann et al., 2012). Dermcidin is bactericidal to *S. epidermidis in vitro* (Liu et al., 2025), and exposure induces production of extracellular proteases (Lai et al., 2007; Paulmann et al., 2012). In *S. epidermidis*, the metalloprotease SepA contributes to proteolysis and is required for accumulation associated protein (Aap)-dependent biofilm maturation (Paharik et al., 2017). Biofilms further impede antimicrobial penetration, with extracellular DNA (eDNA) creating diffusion and retention barriers that support survival under surface stresses (Doroshenko et al., 2014; Vuong et al., 2004). Consistent with these adaptations, *S. epidermidis* grows robustly in sweat-like media in comparative assays and is enriched at moist, eccrine-influenced skin sites (Grice et al., 2009; Swaney et al., 2023). Extending this logic, *S. epidermidis* also shows relative tolerance to sebaceous antimicrobials, such as sapienic acid (Moran et al., 2017). These adaptations enable *S. epidermidis* to persist under the combined chemical and antimicrobial pressures of eccrine secretions, reinforcing its role as a specialized and resilient colonizer of human skin.

### 3.2 Sebaceous glands

Mammalian sebaceous glands are cutaneous holocrine structures typically associated with hair follicles, forming pilosebaceous units. They secrete sebum, a lipid-rich mixture of triglycerides, wax esters, free fatty acids, cholesterol, cholesteryl esters, and squalene (Picardo et al., 2009). In humans, sebaceous glands are among the most abundant skin appendages, with millions distributed across the body surface. They are present in nearly all regions except the glabrous skin of the palms and soles, with peak densities on the face, scalp, chest, and upper back (Thody and Shuster, 1989). While most are follicle-associated, specialized free sebaceous glands occur at select sites, including the eyelids (Meibomian glands) and areolae (Montgomery glands), reflecting site-specific functional adaptations (Butovich, 2017; Luigi et al., 2023).

Among mammals, sebaceous glands are a key adaptation supporting epidermal barrier function and innate defense (Lovászi et al., 2017; Zouboulis et al., 2022). Comparative analyses reveal substantial variation in sebum composition, reflecting ecological and physiological demands (Vanderwolf et al., 2023). Human sebum is notably enriched in sapienic acid (16:1Δ6), generated from palmitic acid by Δ6-desaturase (FADS2) (Ge et al., 2003; Pappas, 2009). This monounsaturated fatty acid appears uncommon in most nonhuman mammals and may represent a human-biased antimicrobial adaptation that helps modulate the human skin microbiome. In humans, free fatty acids constitute ∼15%–30% of total sebum, with sapienic acid comprising ∼20%–25% of this fraction (Cao et al., 2022; Picardo et al., 2009). Sapienic acid exhibits in vitro antibacterial activity, particularly against Gram-positive organisms, and effectively inhibits *S. aureus* (Drake et al., 2008; Fischer et al., 2014; Watanabe et al., 2019). While it antagonizes many pathogens, *S. epidermidis* shows relative tolerance to this lipid-rich milieu (Moran et al., 2017), consistent with its frequent abundance at sebaceous sites.

Mechanistically, sapienic acid disrupts bacterial membranes by inserting into lipid bilayers, altering their structure and fluidity (Parsons et al., 2012). *In vitro*, sapienic acid demonstrates activity against several Gram-positive pathogens, including *S. aureus* (Watanabe et al., 2019). In *S. aureus*, exposure can suppress virulence determinants such as protein A and α-hemolysin (Clarke et al., 2007; Douglas et al., 2025). By contrast, *S. epidermidis* displays relative tolerance to sapienic acid. Experimentally, *S. epidermidis* activates ammonia-generating pathways such as ADI and urease that buffer acidity (Lindgren et al., 2014; Moran et al., 2017). The bacterium also upregulates transporters and engages fatty-acid kinase subunit B2 (fakB2)-mediated incorporation of host unsaturated fatty acids, consistent with membrane remodeling, and produces fatty acid–modifying enzyme (FAME), which detoxifies bactericidal lipids by esterifying them to cholesterol (Chamberlain and Brueggemann, 1997; Moran et al., 2017). The ability to extensively reconfigure its membrane lipid composition by incorporating preformed host fatty acids is hypothesized to further enhance survival in the lipid-rich cutaneous environment (Tiwari et al., 2020). Collectively, these adaptive traits likely confer a selective advantage in sebaceous niches, enabling *S. epidermidis* to persist where more sensitive pathogens are excluded.

Sebaceous glands also support a symbiotic relationship with lipophilic *Malassezia* spp., which rely on sebum-derived lipids for growth (Wu et al., 2015). *Malassezia* produce bioactive metabolites, such as tryptophan-derived indoles, that activate the aryl hydrocarbon receptor (AhR) in skin immune cells, thereby contributing to immune homeostasis and modulating inflammatory responses (Vlachos et al., 2012). In keratinocytes, *Malassezia furfur* activates Toll-like receptor 2 (TLR2), initiating intracellular signaling that promotes proinflammatory mediator release (Baroni et al., 2006). Likewise, *Malassezia globosa* and *Malassezia restricta* stimulate keratinocyte expression of TLR2, interleukin (IL)-8, and AMPs, enhancing local innate defenses (Donnarumma et al., 2014). Beyond immune modulation, *M. globosa* also secretes an aspartyl protease (MgSAP1) that hydrolyzes *S. aureus* protein A and attenuates biofilm formation, thereby directly antagonizing a major cutaneous pathogen (Li et al., 2018). *In vivo*, *Malassezia* spp. elicit a robust IL-17-mediated immune response characterized by expansion of CCR6^+^ Th17 T cells. This response is pivotal for controlling fungal colonization and orchestrating antifungal immunity, although it may exacerbate skin inflammation when the epithelial barrier is impaired (Sparber et al., 2019).

Beyond these roles, it has been hypothesized that *Malassezia* spp. contribute to human anti-acarian defense (Retzinger and Retzinger, 2024b,c). Indirect evidence lends plausibility to this idea, though it remains circumstantial. First, other basidiomycetes such as *Meira* exhibit anti-mite activity in plant hosts, providing an ecological analogy rather than a direct mammalian precedent (Gerson et al., 2008). Second, epidemiological studies suggest that IgE-mediated allergic disease peaks before puberty, a developmental stage characterized by low sebaceous activity and limited *Malassezia* colonization (Hill et al., 2016; Katsanakis et al., 2024). Third, per the Acari Hypothesis, the IgE targeting of *Malassezia*-derived proteins in atopic individuals implies deleterious effects of these fungi on acarian physiology (Brodská et al., 2014; Glatz et al., 2015; Retzinger and Retzinger, 2024b). Contributions of *Malassezia* spp. to mammalian defense have not yet been experimentally demonstrated, but collectively these observations suggest that sebaceous gland–associated *Malassezia* may protect against acarians. If such a role exists, *Malassezia* and *S. epidermidis* could act cooperatively as part of a broader microbial consortium favored by sebaceous secretions, rather than as isolated defensive symbionts. If eccrine and sebaceous glands generate cutaneous ecosystems that select for microbes conferring defense against pathogens and ectoparasites, then apocrine glands may represent an analogous system contributing to resistance against acarians.

### 3.3 Apocrine glands

In many mammalian species, apocrine glands are key exocrine structures for chemical communication, mediating social signaling, territorial marking, and reproductive behaviors through pheromonal compounds (Fontani et al., 2022; Janssenswillen et al., 2021; Kioko et al., 2017; Leclaire et al., 2017; Ma et al., 2022; Svendsen and Jollick, 1978; Theis et al., 2012). These glands release their products via apocrine secretion, a process in which the apical portion of the secretory cell pinches off, releasing cytoplasmic content and membrane fragments together with secretory products (Gesase and Satoh, 2003). This mechanism delivers complex mixtures of lipids, proteins, and other bioactive molecules that establish a distinctive microenvironment for resident microbes.

In humans, the role of apocrine glands remains incompletely defined. Comparative evidence indicates that the communicative function of apocrine glands in other mammals often depends on microbial metabolism: apocrine secretions acquire full biological activity only after bacterial conversion (Leclaire et al., 2017; Ma et al., 2022; Theis et al., 2012). Because microbial processing is essential, the ancestral evolutionary role of apocrine glands may have extended beyond communication to include maintenance of specialized cutaneous niches that support metabolically active microbiota. From this perspective, human apocrine glands may retain physiological relevance by fostering microbial partners whose functions extend beyond chemical signaling to additional roles, such as defense against pathogens and ectoparasites. In the axilla, for example, *Staphylococcus* spp. (notably *S. epidermidis* and *S. hominis*) and *Corynebacterium* spp. metabolize odorless precursors, sulfur-containing amino acids and short-chain fatty acid conjugates, into volatile compounds that generate the characteristic odors of apocrine sites (Hara et al., 2014; Lam et al., 2018; Leyden et al., 1981; Mogilnicka et al., 2020; Natsch et al., 2003; Troccaz et al., 2015).

Comparative insights from the Western honeybee (*Apis mellifera*) and its hive-associated microbiota provide an analogy, illustrating how microbial volatiles can contribute to host defense. Worker bees synthesize 2-heptanone in their mandibular glands and release it when biting ectoparasitic mites, inducing transient paralysis that facilitates mite removal (Papachristoforou et al., 2012). Beyond its antiparasitic action, 2-heptanone also functions as a chemical signaling molecule (alarm pheromone) in *A. mellifera*, coordinating nestmate communication and defensive recruitment (Balderrama et al., 2002). 2-heptanone is also generated by hive-associated microbes (Saccà et al., 2021). This analogy suggests that volatile compounds may originate in antiparasitic defense, with communicative roles emerging secondarily.

Human apocrine glands, concentrated in the axillae and inguinal/perineal regions [with specialized apocrine variants at selected sites such as the nasal vestibule, eyelid (Moll’s glands), and external auditory canal (ceruminous glands)], harbor microbial communities enriched in *Staphylococcus* and *Corynebacterium* (Callewaert et al., 2013; Oh et al., 2014; Petrillo et al., 2020; Rawls and Ellis, 2019; Sjövall et al., 2021). Reported volatile organic compounds (VOCs) produced by *S. epidermidis* include acetic acid, isovaleric acid, 2-methylbutanal, 3-methylbutanal, and 5-methyl-2-heptanone, a structural analog of bee-derived 2-heptanone (Jenkins and Bean, 2019; Mayer et al., 2021). The functional significance of these VOCs in humans remains uncertain. However, given established defensive roles of analogous volatiles in other taxa, it is plausible that they may contribute to protection from ectoparasites or other pathogens, independent of any role in olfactory signaling. To date, there have been no studies directly measuring the biochemical responses of human integumentary glands or their resident microbes to acarian infestation.

In summation, these observations support a model in which apocrine glands actively contribute to host defense and microbiota maintenance. By fostering microbial communities that generate bioactive metabolites, apocrine glands may influence the cutaneous ecosystem and enhance host fitness. This framework also offers a lens on mammary-gland evolution: if apocrine glands are adapted to support specific symbionts, glandular function in mammalian progeny would be compromised unless offspring reliably acquire those microbes. In this context, mammary glands likely evolved to promote vertical transmission of microbiota from mother to progeny, ensuring that neonatal epithelial surfaces are effectively seeded with beneficial symbionts.

### 3.4 Mammary glands

Mammary glands are a defining synapomorphy of mammals (Oftedal, 2002). Believed to derive from apocrine glands, mammary glands exhibit substantial anatomical and functional diversity across extant lineages. In monotremes (e.g., platypus, echidna), milk is secreted onto a nipple-less areolar mammary patch with specialized hairs, and the young lap milk from the hair coat (Griffiths, 1983). Marsupials and eutherians possess nipples supplied by lactiferous ducts, enabling direct transfer during suckling. Eutherian mammary glands are typically more elaborate, with well-developed ductal–alveolar systems that support efficient, sustained lactation (Oftedal, 2002). Human mammary glands consist of lobuloalveolar units where milk is synthesized by secretory lactocytes. Myoepithelial cells surrounding the alveoli propel milk into a branched ductal network during let-down. These ducts converge into larger lactiferous ducts that open at the nipple. Although the breast is classically described as having ∼15–20 lobes, imaging studies show considerable variability, with typically ∼5–9 major duct orifices per nipple (Love and Barsky, 2004).

Beyond nutrition, human milk provides a coordinated immune-microbial defense in early life. It delivers secretory IgA, lactoferrin, lysozyme, immunomodulatory cytokines (e.g., transforming growth factor-β, IL-10), and human milk oligosaccharides (HMOs) that sculpt infant microbial communities (Agarwal et al., 2011; Al-Beltagi, 2025; Rio-Aige et al., 2021). Milk also consistently contains commensal bacteria, e.g. *Staphylococcus*, *Streptococcus*, *Lactobacillus*, and *Bifidobacterium*, that seed the infant gastrointestinal tract (Edwards et al., 2022; Fitzstevens et al., 2017; Ojo-Okunola et al., 2018; Williams et al., 2017). In addition to inputs from skin, an entero-mammary pathway now appears to be an important source of these microbes: maternal immune cells sample gut commensals, traffic them via lymphatic and/or blood routes to the mammary gland, and release them into milk, enabling targeted, strain-level transfer to the neonate (Rodríguez, 2014). Together, these features indicate that the milk microbiota is a regulated component of maternal-offspring biology rather than incidental contamination.

Experimental studies in mice and humans support an entero-mammary route. In lactating mice, orally administered fluorescently labeled *Lactobacillus* have been recovered from mammary tissue and milk, consistent with gut-to-gland trafficking (de Andrés et al., 2017). Mechanistically, dendritic cells and mononuclear phagocytes have been proposed to facilitate the entero-mammary pathway by sampling bacteria in the gut and trafficking them to the mammary gland; supporting evidence includes bovine and human studies detecting bacterial DNA from gut-associated taxa within circulating leukocytes and milk somatic cells (Perez et al., 2007; Young et al., 2015). In humans, culture-based and molecular studies likewise support a gut-to-mammary axis. Viable *Lactobacillus* strains administered orally to lactating women have subsequently been isolated from breast milk, in the absence of cutaneous or oral contamination, suggesting systemic trafficking rather than external transfer (Jiménez et al., 2008b). Detection of identical strains in maternal feces and milk further reinforces this interpretation (Zhang et al., 2020). Together, these findings across model organisms and humans converge on an entero-mammary pathway in which maternal immune cells serve as vectors for selected commensals from the gut to the gland.

In human breast milk, the most consistently detected genera are *Staphylococcus* and *Streptococcus* (Zhang et al., 2020), with *S. epidermidis* often the dominant staphylococcal species across cohorts and regions (Heikkilä and Saris, 2003; Ingram et al., 2024). As with skin, the milk/mammary niche is rich in antimicrobials (Schlievert et al., 2019; Sprong et al., 2001; Trend et al., 2015), yet *S. epidermidis* persists, outcompeting pathogens like *S. aureus* in mammary secretions (Heikkilä and Saris, 2003), supporting the view that *S. epidermidis* is unusually well adapted to the human mammary epithelial microenvironment.

Human milk also conveys fungi to the neonate, and the nipple–areola complex helps stage that transfer. Around the areola, Montgomery glands, large, modified sebaceous units, secrete a lipid-rich, antimicrobial film that lubricates and protects the nipple and likely supplies olfactory cues for the newborn (Doucet et al., 2009; Luigi et al., 2023; Montagna and Yun, 1972). Most Montgomery glands are accompanied by a short lactiferous duct. Their sebaceous ducts open onto the areolar surface or merge with this short duct just before it reaches the epidermis. Although distinct from the primary milk ducts, this arrangement positions sebaceous secretions adjacent to ductal orifices during latch. In parallel, culture-independent surveys and select culture studies detect *Malassezia*, most often *M. globosa* and *M. restricta*, in colostrum and mature milk, indicating that milk itself can serve as a fungal source (Boix-Amorós et al., 2017, 2019). Together with the areolar interface, this likely facilitates vertical transmission of *Malassezia*. Consistent with this, *Malassezia* appears on most neonates within hours to days of birth, and early strain profiles often mirror maternal skin communities (Nagata et al., 2012). Breastfeeding is also associated, across multiple cohorts, with reduced allergy risk (Chiu et al., 2016; Ding et al., 2024; Hou et al., 2024; Lodge et al., 2015). Although mechanisms are likely multifactorial, it is tempting to speculate that milk-borne microorganisms are part of this protective effect.

Multiple cohort studies and meta-analyses associate breastfeeding with a modest reduction in allergic disease. Breastfed infants also tend to acquire maternal skin–associated taxa earlier, notably *S. epidermidis* and *Malassezia*, a pattern that in several cohorts tracks with reduced allergic morbidity (Stewart et al., 2018; van den Elsen et al., 2019). Within the Acari Hypothesis, such vertical transfer could bolster defenses relevant to ectoparasite exposure, offering a plausible mechanism for part of breastfeeding’s allergy benefit. These observations point toward a broader possibility: that *S. epidermidis* functions as a human mutualist.

### 3.5 Developmental ecology of human integumentary glands

While eccrine, sebaceous, apocrine, and mammary glands are often considered as discrete anatomical entities, their functions are ecologically interdependent and coordinated across organism maturation. Viewed through the lens of the proposed apocrine-mammary-microbiota axis, these glands collectively define a dynamic, stage-specific system that shapes the establishment, selection, and maintenance of the human cutaneous microbiota across the lifespan.

Sebaceous glands are highly active during late gestation under the influence of maternal and placental androgens (Zouboulis, 2010), contributing to the formation of vernix caseosa, a lipid-rich biofilm composed of sebaceous secretions and desquamated corneocytes (Nishijima et al., 2019). Vernix, a feature most prominently developed in humans, coats the neonatal skin at birth, supporting barrier function while shaping the early cutaneous microenvironment and likely influencing initial microbial colonization. In this context, sebaceous gland activity provides an early lipid-rich niche that may facilitate the establishment of microbial communities, particularly within pilosebaceous units.

In contrast, eccrine glands are fully formed at birth and, owing to the relatively small body surface area of infants, are present at high density during early life (Baker, 2019). Consequently, eccrine secretions are likely to dominate the skin microenvironment immediately after birth. This establishes a distinct ecological phase in which microbial communities must adapt to a unique, chemically restrictive milieu. Eccrine activity may therefore play a central role in regulating microbial composition, selecting for taxa capable of tolerating fluctuating pH and host-derived antimicrobials.

Apocrine glands, while anatomically present at birth, remain largely inactive until puberty (Beier et al., 2005). Nevertheless, during vaginal delivery, neonates are exposed to maternal skin and mucosal secretions from apocrine-rich anogenital regions, providing a complex mixture of microbial and biochemical inputs (Dominguez-Bello et al., 2010). Although the specific contribution of maternal apocrine-derived secretions to neonatal colonization has not been directly defined, they likely shape early niche conditions and microbial transfer. Following pubertal maturation, the individual’s own apocrine glands assume a prominent role, generating specialized microenvironments that support dense, metabolically active microbial communities, including staphylococci and corynebacteria.

Together, these observations support a developmentally staged model of cutaneous ecology: an initial sebaceous-dominated phase associated with vernix and early microbial establishment; a subsequent eccrine-dominated phase that regulates microbial selection and environmental stability throughout infancy and childhood; and a later apocrine-associated phase that expands niche complexity and supports specialized microbial functions in adulthood. Within this framework, the mammary gland extends these processes across generations by enabling the vertical transfer of maternal microbiota, thereby reinforcing continuity of host-microbe interactions.

Epidemiologic studies provide indirect support for the importance of early-life microbial exposures in shaping long-term health. Cesarean delivery is consistently associated with increased risk of immune-mediated and metabolic disorders, including atopic disease, asthma, and obesity, among others (Blustein and Liu, 2015; Chavarro et al., 2020; Darabi et al., 2019; Sevelsted et al., 2015). These associations are widely attributed to altered microbial colonization, as infants delivered vaginally are exposed to maternal vaginal and perineal microbiota, whereas those delivered by Cesarean section acquire communities more reflective of skin and environmental sources. Within this context, the maternal anogenital region, enriched in apocrine glands, represents a complex biochemical and microbial environment to which the neonate is exposed during vaginal delivery. While the specific contribution of apocrine-derived secretions remains to be defined, their absence in Cesarean delivery may reflect not only a shift in microbial exposure but also a loss of glandular-derived biochemical inputs that have co-evolved with host-microbe interactions.

This developmental-ecological framework provides a foundation for understanding how key members of the human cutaneous microbiota, particularly *S. epidermidis*, are established, maintained, and functionally integrated into host physiology. In the following section, the role of *S. epidermidis* as a mutualistic partner in epithelial defense, immune modulation, and metabolic regulation is examined within this broader context.

## 4 Mutualism between *S. epidermidis* and *Homo sapiens*

*S. epidermidis* is highly adapted to the human skin environment, with growing evidence that it functions primarily as a host-associated bacterium reliant on epithelial surfaces for persistence (Grice et al., 2009; Kloos and Musselwhite, 1975; Kloos, 1980; Oh et al., 2014). Reports of its occurrence on primates outside humans are uncommon, and when identified in domestic animals, hospital environments, or laboratory settings, it is often attributed to transient carriage or anthropogenic contamination. Comparative sampling of non-human primate skin in captivity found *S. epidermidis* to be rarely isolated (Wildsmith et al., 2025). Most staphylococcal isolates on nonhuman primates belonged to other species, supporting the idea that reports of *S. epidermidis* outside humans frequently reflect transient or contamination events. Unlike many free-living bacteria, *S. epidermidis* relies on a specialized, host-associated metabolism rather than broad saprophytic versatility. This dependency may have been reinforced early in mammalian evolution through maternal transmission, potentially mediated by the mammary gland, thereby promoting stable colonization across generations.

Co-evolution of *S. epidermidis* with human epithelial surfaces has important consequences for both the bacterium and its host, with evidence suggesting the bacterium contributes actively to epithelial homeostasis. Recent work has shown that commensal-specific T cells induced by skin microbiota exhibit functional plasticity, rapidly adopting IL-17A–producing phenotypes in response to tissue injury and promoting barrier repair and homeostasis (Harrison et al., 2019). Mechanistically, these responses arise through antigen presentation by cutaneous dendritic cells, which sample commensal-derived antigens and drive the expansion of tissue-resident T cell populations within the skin. These commensal-specific T cells persist locally and, upon epithelial disruption, are rapidly reactivated to produce cytokines such as IL-17A, which enhance antimicrobial peptide production, recruit neutrophils, and stimulate keratinocyte proliferation and differentiation. In this way, *S. epidermidis* contributes not only to epithelial integrity through direct interactions with keratinocytes, but also through the programming of adaptive immune responses that reinforce tissue resilience.

*S. epidermidis* also modulates keratinocyte activity directly. *S. epidermidis* LTA engages TLR2, which suppresses excessive inflammation and accelerates re-epithelialization after injury, thereby enhancing repair while preventing tissue damage from uncontrolled immune activation (Lai et al., 2009). Colonization by *S. epidermidis* has also been shown to improve barrier integrity in murine models of dermatitis, reducing transepidermal water loss and reinforcing tight junction proteins such as claudin-1 and occludin (Masuda-Kuroki et al., 2023; Ohnemus et al., 2008). In parallel, *S. epidermidis* promotes keratinocyte lipid synthesis, including ceramides and free fatty acids essential for stratum corneum integrity, while also inducing AMPs such as β-defensins that fortify the chemical barrier (Lai et al., 2010; Zheng et al., 2022). When the epidermal barrier is breached, *S. epidermidis* can modify keratinocyte differentiation and proliferation, suggestive of a barrier-reparative response (Duckney et al., 2013). *S. epidermidis* has also been shown to stimulate collagen type I production in dermal fibroblasts through free fatty acid receptor 2 (FFAR2)/phosphorylated extracellular signal-regulated kinases (p-ERK) signaling, underscoring its capacity to actively reinforce structural components of the skin barrier in addition to modulating keratinocyte responses (Negari et al., 2021). Collectively, these findings support a model in which *S. epidermidis* functions as a mutualistic partner, contributing to the maintenance and restoration of human epithelial integrity.

In parallel with reinforcing the human epithelial barrier, many *S. epidermidis* strains also maintain their own community barrier through biofilm formation. Biofilms represent a core physiological strategy that benefits the bacterium and potentially benefits the host. The extracellular matrix of polysaccharides, proteins, and extracellular DNA provides a stable microenvironment that sequesters nutritive macromolecules secreted by epithelial cells, concentrating nutrients that would otherwise diffuse away (Otto, 2009). The biofilm also shields the bacterium from desquamation, washing, and host immune clearance, ensuring long-term persistence on skin (Paharik et al., 2017). Importantly, biofilm communities can antagonize pathogens: the matrix limits pathogen access to epithelial surfaces, while resident S. *epidermidis* secretes AMPs and phenol-soluble modulins (PSMs) that inhibit competitors such as *S. aureus* (Cogen et al., 2010). However, the contributions of PSMs are dependent on context. While they contribute to pathogen suppression and biofilm structuring, certain PSMs can also trigger inflammation or cytotoxic effects when dysregulated (Otto, 2013). This duality underscores the delicate balance between mutualism and pathogenicity in *S. epidermidis* biofilm physiology.

Carbohydrates play a central role in biofilm formation. *S. epidermidis* biofilms are stabilized by polysaccharide intercellular adhesin (PIA), a β-1,6-linked N-acetylglucosamine polymer encoded by the icaADBC operon, with UDP-N-acetylglucosamine as the immediate precursor (Rohde et al., 2010). Glucose availability strongly influences PIA production and biofilm density: elevated glucose enhances PIA synthesis and drives robust biofilm formation (Agarwal and Jain, 2013; Arciola et al., 2015). Genome-scale metabolic modeling of a representative strain of *S. epidermidis* (RP62A) suggests that a substantial fraction of glucose flux is diverted away from glycolysis and toward UDP-N-acetylglucosamine biosynthesis and other matrix-related pathways (Díaz Calvo et al., 2022). Complementing this, experimental studies show that under high-glucose conditions *S. epidermidis* redirects carbon to the amino-sugar pathway and PIA synthesis, a shift accentuated under nitrosative stress (Oliveira et al., 2023). Together, these findings suggest that, for at least some *S. epidermidis* populations, glucose serves less as a growth substrate and more as a structural resource for community stability and persistence. From an ecological standpoint, this metabolic strategy highlights how skin nutrient landscapes directly shape *S. epidermidis* physiology. From a co-evolutionary perspective, it is possible that human skin physiology influences surface glucose availability in ways that promote biofilm formation and sustain symbiotic populations. In this way, the skin barrier is not a passive substrate but an actively structured habitat that supports biofilm-forming symbionts like *S. epidermidis*.

This metabolic interdependence may extend beyond the skin. Under tryptophan-rich conditions, *S. epidermidis* can generate trace biogenic amines, including serotonin (5-hydroxytryptamine), through alternative decarboxylation pathways, although the physiological significance of this remains to be fully defined (Luqman et al., 2018, 2020). In mammals, serotonin plays a well-established role in metabolic regulation: in pancreatic β-cells, it enhances insulin synthesis and secretion, and in enteroendocrine L-cells, it promotes glucagon-like peptide-1 (GLP-1) release, thereby potentiating insulin signaling (Paulmann et al., 2009; Ripken et al., 2016). These observations raise the possibility *that S. epidermidis* may influence host metabolism indirectly through modulation of serotonergic signaling, particularly at epithelial interfaces where microbial metabolites can access host endocrine pathways.

A previous installment of the Acari Hypothesis proposed a role for *S. aureus* in metabolic syndrome and cardiovascular disease (Retzinger and Retzinger, 2025). Certain *S. epidermidis* strains inhibit *S. aureus* colonization, indicating a possible protective role (Nakatsuji et al., 2017; Sandiford and Upton, 2012; Suzuki et al., 2024). Beyond colonization resistance, emerging evidence suggests that *S. epidermidis* may also directly influence incretin biology. In a high-throughput functional screen of approximately 1500 human-derived bacterial isolates, only 45 strains were found to increase GLP-1 secretion in enteroendocrine models; notably, all GLP-1-stimulatory isolates were identified as *S. epidermidis* (Tomaro-Duchesneau et al., 2020). This striking enrichment suggests that GLP-1-modulating activity may represent a conserved or widely distributed functional characteristic of the species rather than a rare strain-specific property. Mechanistically*, S. epidermidis* secretes a small peptide, GLP-1 stimulatory peptide A (GspA), also identified as a specific form of delta-toxin, capable of stimulating GLP-1 release in an in vitro model, through direct interaction with enteroendocrine L cells and activation of intracellular signaling pathways governing incretin secretion. In contrast, other members of the microbiota may exert opposing effects on this axis: *Enterococcus faecalis* produces a secreted metalloprotease (GelE) that degrades GLP-1, thereby reducing its bioavailability and attenuating downstream metabolic signaling (LeValley et al., 2020).

The identification of *S. epidermidis* as a dominant GLP-1-stimulatory organism is unexpected, as it is not classically regarded as a stable colonizer of the gastrointestinal tract. However, GLP-1 is produced by enteroendocrine L cells localized to the intestinal epithelium (Tolhurst et al., 2009), implying that for this interaction to have physiological relevance, *S. epidermidis* or its secreted products must at least transiently access the gut lumen or mucosal surface. Notably, *S. epidermidis* is detected in substantial abundance in the stool of breastfed infants, consistent with early-life exposure and potential gastrointestinal transit or colonization (Jiménez et al., 2008a). It is therefore plausible that discrete populations established during infancy persist in protected or low-abundance niches within the gastrointestinal tract across the lifespan, where they could intermittently influence host GLP-1 signaling pathways.

These mechanistic observations are supported by broader clinical and translational data linking microbiota composition to GLP-1 dynamics and metabolic disease. Alterations in gut microbial communities in gestational diabetes mellitus have been correlated with circulating GLP-1 levels, with specific taxa (including *Sutterella*, *Oscillibacter*, and *Bifidobacterium*) showing positive associations with GLP-1–related measures (Liang et al., 2022). In parallel, systematic screening frameworks for probiotic candidates in obesity management increasingly incorporate the capacity to modulate host metabolic pathways including incretin signaling, alongside effects on inflammation, epithelial barrier function, and nutrient metabolism (Alard et al., 2021). Together, these findings support a model in which microbial regulation of GLP-1 is functionally significant. Because pharmacologic GLP-1 receptor agonism confers substantial benefit in metabolic syndrome, obesity, and type 2 diabetes (Astrup et al., 2012; Sandsdal et al., 2023), insufficient colonization by GLP-1–stimulatory commensals such as *S. epidermidis*, or overrepresentation of GLP-1–degrading organisms such as *E. faecalis*, may represent a plausible microbiome-linked contributor to metabolic disease risk.

Despite its many host benefits, *S. epidermidis* has long been classified as an opportunistic pathogen due to its frequent association with nosocomial infections, particularly those involving indwelling medical devices (Otto, 2009; Widerström, 2016). These infections typically rely on biofilm formation mediated by the icaADBC operon and PIA. Importantly, they rarely involve intact tissue, and the inflammatory response attributed to *S. epidermidis* often resolves once the device is removed (McCann et al., 2008). Given the proposed evolutionary alignment between humans and *S. epidermidis* and the bacterium’s demonstrated contributions to barrier maintenance and repair, its role in nosocomial contexts warrants re-evaluation. Rather than true opportunistic invasion, such infections may represent an immune response to a foreign surface, one that abates only when the device is eliminated, and epithelial integrity restored. From this perspective, *S. epidermidis* may be better understood as a sentinel mutualist, mobilizing host defenses when epithelial integrity is compromised, rather than a classical pathogen.

This sentinel model underscores the broader contention that *S. epidermidis* may be essential for maintaining cutaneous homeostasis. By simultaneously reinforcing the skin barrier and buttressing host antimicrobial defenses, the bacterium plays a central role in epithelial resilience. However, this equilibrium is not immutable. When *S. epidermidis* populations are diminished or functionally impaired, as might occur through formula supplementation, the hygienic disruption of the epithelial microenvironment, or the use of antimicrobials, the stability of the cutaneous ecosystem collapses. Loss of protective strains compromises barrier integrity, reduces antimicrobial defense, and permits the overgrowth of more pathogenic taxa.

The dependence of *S. epidermidis* on human epithelial surfaces, its abundance in human mammary secretions, its evolved tolerance of endogenous antimicrobials, and its demonstrated contributions to cutaneous and metabolic fitness suggest that this species is more than a passive commensal, it is better understood as a fundamental mutualist. Within its native human epithelial ecosystem, *S. epidermidis* suppresses competitors and restricts the expansion of potential pathogens. Accordingly, deficiency in *S. epidermidis* may generate two categories of clinical consequences. The first are “negative” symptoms, reflecting inefficiencies in epithelial repair or metabolic regulation due to the absence of mutualistic functions. The second are “positive” symptoms, which arise when ecological vacancies are filled by pathogenic staphylococci or other opportunists. The latter is supported by the growing recognition that reduced *S. epidermidis* abundance or activity facilitates pathogen colonization and contributes to diverse epithelial disorders.

Staphylococcal dysbiosis has emerged as a defining feature of modern dermatopathology. Across conditions such as atopic dermatitis, psoriasis, hidradenitis suppurativa, cutaneous lupus erythematosus, chronic wounds, acne, and seborrheic dermatitis, a consistent pattern is observed: depletion of protective *S. epidermidis* and enrichment of pro-inflammatory or pathogenic species including *S. aureus*, *Staphylococcus lugdunensis*, and, in some reports, *Staphylococcus pettenkoferi* (Cucu et al., 2025; James et al., 2008; Kim et al., 2025; Lee et al., 2019, 2025; Loesche et al., 2017; Sirobhushanam et al., 2020; Tamer et al., 2018). Pathogenic staphylococci may not directly cause disease, but their presence often signals the loss of commensal control and an ecosystem vulnerable to invasion. Loss of *S. epidermidis* undermines cutaneous homeostasis by diminishing antimicrobial defense, weakening barrier integrity, and removing quorum-sensing interference that normally restrains virulence in competing taxa. Conversely, expansion of pathogenic staphylococci introduces cytotoxins, superantigens, and pro-inflammatory cell wall components that amplify immune activation, disrupt keratinocyte differentiation, and sustain chronic inflammation. These shifts are increasingly recognized not as secondary consequences of skin disease but as active drivers of onset and persistence, reframing common dermatological disorders as manifestations of microbial imbalance. From this perspective, restoring *S. epidermidis* populations or their functional outputs represents a promising foundation for microbiome-based therapies.

## 5 Closing

Despite advances in microbiome research, our understanding of the prehygiene human microbiome remains limited. Modern practices, e.g., partial or complete replacement of breast milk with infant formula, widespread antibiotic use, and hygiene-driven alteration of glandular secretions, have likely reshaped the epithelial microbiota. Reconstructions based on isolated pre-industrial populations, ancient DNA, and nonhuman primate comparisons are constrained by sparse sampling, methodological heterogeneity, and ecological non-equivalence, leaving the composition and diversity of ancestral communities uncertain. Accordingly, any portrait of a “natural” human microbiota should be interpreted cautiously and contextually, acknowledging the dynamic, contingent nature of host–microbe interactions over evolutionary time.

Within this uncertainty lies another layer of complexity: the human microbiome has never been uniform, even in ancestral contexts. Historical population structure and local ecologies produced differences in integumentary phenotypes, from skin pigmentation and sebum profiles to hair architecture and sweat gland activity, that in turn shape the skin microbiota. Diversity is especially evident within the genus *Malassezia*, whose species distributions vary across skin types. For example, several studies report higher prevalence of *M. restricta* and *M. globosa* in some East Asian cohorts, with *M. japonica*, first isolated from healthy Japanese skin, more often detected in East Asian skin ecosystems (Gaitanis et al., 2012; Sugita et al., 2003, 2010; Xie et al., 2014). *Malassezia slooffiae*, while global, appears at elevated prevalence in certain East Asian samples (Kaneko et al., 2010), whereas *Malassezia sympodialis* is frequently dominant in parts of Europe and North America (Jagielski et al., 2014). These patterns likely reflect co-evolution with host lipid profiles, immune traits, and environmental exposures, while acknowledging substantial within-group variability and methodological heterogeneity.

This geographic and genetic diversity raises questions about the consequences of admixture among populations with differing skin phenotypes and locally adapted microbiomes. For example, an infant whose inherited integumentary traits differ from those of the primary microbial donor in early life may acquire *Malassezia* and other taxa not optimally adapted to that skin environment. Such phenotype–microbiome incongruence could perturb host–microbe homeostasis and increase vulnerability to barrier dysfunction or inflammation. Unraveling these interactions will require longitudinal, integrative studies across ethnically and geographically diverse cohorts, with careful control for shared environment and socioeconomic confounders.

Although *S. epidermidis* is likely a keystone symbiont of human skin, a broader consortium of staphylococci potentially contributes to host mutualism within the apocrine–mammary–microbiota axis. For example, *Staphylococcus auricularis*, closely associated with the ceruminous glands of the external auditory canal, may support epithelial homeostasis in that niche (Kloos and Schleifer, 1983). Likewise, the ocular surface includes modified apocrine glands (glands of Moll) along the eyelid margin that shape the local tear film microenvironment (Stoeckelhuber et al., 2003). These specialized apocrine structures, like their inguinal and axillary counterparts, provide distinctive biochemical niches that foster specific microbial partnerships and may have co-evolved with particular lineages to sustain tissue-specific functions.

Finally, efforts to genetically engineer components of the human microbiota raise ethical challenges. Because *S. epidermidis* is vertically transmitted from mother to child and readily exchanged through close contact, engineered strains could spread through human populations as transmissible, heritable modifications, functionally analogous to germline mutations. Such interventions must therefore be approached with rigorous ecological and ethical scrutiny.

To move beyond conceptual synthesis toward empirical validation, a central strength of a hypothesis framework lies in its capacity to generate falsifiable predictions. The apocrine–mammary–microbiota axis proposed here yields several testable hypotheses. First, if apocrine secretions actively select for *S. epidermidis*, then reconstructed human skin systems supplemented with apocrine-derived secretions should demonstrate selective enrichment of *S. epidermidis* relative to other staphylococci, whereas depletion or chemical alteration of these secretions should reduce its competitive fitness. Second, spatially resolved microbiome profiling combined with IgA-seq or related immune-targeting approaches should reveal preferential host interaction with *S. epidermidis* at apocrine-rich sites, consistent with active host selection rather than passive colonization. Third, within human populations, variation in integumentary gland density, activity, or secretion chemistry should correlate with differences in *S. epidermidis* abundance, strain composition, and functional output, providing a natural experiment for testing gland–microbe coupling. Fourth, perturbation studies predict that disruption of early-life microbial transmission, through formula feeding, antibiotic exposure, or altered maternal microbiota, should measurably impair establishment of *S. epidermidis* and be associated with downstream changes in epithelial barrier function, immune calibration, or microbial community stability. Finally, the hypothesis is falsified if apocrine secretions do not reproducibly enrich for *S. epidermidis* in controlled systems, if its distribution is independent of glandular architecture within humans, or if depletion of *S. epidermidis* fails to produce measurable effects on epithelial defense, microbial competition, or host physiology. Together, these predictions define a tractable experimental agenda capable of validating or refuting the proposed axis.

In sum, the proposed mammalian apocrine–mammary–microbiota axis suggests that microbial symbiosis is fundamentally integrated into mammalian evolutionary architecture. In modern humans, this finely balanced system is increasingly perturbed by contemporary societal and medical practices. Such disruptions may impact immunity, development, and even intergenerational health. Recognizing the microbiome not as an accessory but as an inherited component of human biology reframes the responsibilities and objectives of modern medicine: interventions in this ecosystem, whether clinical or social, must be pursued with a respect for the underlying mammalian evolutionary design.

## Data Availability

The original contributions presented in this study are included in the article/supplementary material, further inquiries can be directed to the corresponding author.
